# Pulse Ultrasonic Cure Monitoring of the Pultrusion Process

**DOI:** 10.3390/s18103332

**Published:** 2018-10-05

**Authors:** Patrick Scholle, Michael Sinapius

**Affiliations:** Institute of Adaptronics and Function Integration, Technische Universität Braunschweig, 38106 Braunschweig, Germany; m.sinapius@tu-braunschweig.de

**Keywords:** CFRP, pulse ultrasonic method, cure monitoring

## Abstract

This article discusses the results of a series of experiments on pulse ultrasonic cure monitoring of carbon fiber reinforced plastics applied to the pultrusion process. The aim of this study is to validate the hypothesis that pulse ultrasonic cure monitoring can be applied (a) for profiles having small cross sections such as 7 mm × 0.5 mm and (b) within the environment of the pultrusion process. Ultrasonic transducers are adhesively bonded to the pultrusion tool as actuators and sensors. The time-of-flight and the amplitude of an ultrasonic wave are analyzed to deduce the current curing state of the epoxy matrix. The experimental results show that ultrasonic cure monitoring is indeed applicable even to very thin cross sections. However, significant challenges can be reported when the techniques are used during the pultrusion process.

## 1. Introduction

Carbon Fiber Reinforced Plastics (CFRPs) have been gaining more and more attention over the years due to their high load bearing capabilities and stiffnesses. Their simultaneously low density makes them very interesting in many lightweight applications. CFRPs are fundamentally built from two parts: fiber and matrix. In many mechanically demanding applications, the matrix is currently a thermosetting polymer that is being cured at elevated temperatures after the part receives its final shape. The success of this curing process is crucial for the mechanical properties of the final part. In an effort to allow the monitoring of the curing process, a variety of different cure monitoring techniques have been developed over the years [1,2].

Many of these techniques can provide very accurate information about the current curing state of the resin, but are not suitable for integration into an industrial manufacturing system. Common examples are the Differential Scanning Calorimetry (DSC), Rheometry, and Fourier Transform Infrared spectrometry (FTIR) [3]. These techniques are not applicable to cure monitoring in manufacturing processes as they typically allow only small samples with small dimensions and require expensive equipment. They are therefore only used for the characterization of resins in a laboratory environment. Other techniques have been developed that enable online cure monitoring in manufacturing processes. A well established method is dielectric cure monitoring [4]. In this technique, a small sensor is placed in contact with the resin. During measurement, the impedance of the sensor is affected by a change in resin viscosity, thus allowing an online measurement of the current curing state. Other methods apply fiber optical instruments. In Fresnel’s reflection fiber optic cure monitoring, the change in the refractive index between a cleaved glass fiber and the resin is monitored during curing [5]. Fiber Bragg Grating (FBG) cure monitoring measures the residual strain development during cure. Other online monitoring approaches measure the temperature in the curing epoxy [6]. Both the peak temperature and the time required to reach this peak can be used as data to determine the current curing state. Most of these online cure monitoring approaches have one thing in common: the need for direct contact between the sensor and the resin. The methods therefore cannot be applied in a highly abrasive environment such as the pultrusion analyzed in this study. Notably, a temperature measurement based on thermography, where the computationally intensive image analysis tools are being sped up consecutively [7], could provide a contact free approach for cure monitoring. However, image acquisition is not compatible with closed mould processes such as pultrusion.

**Figure 1:**
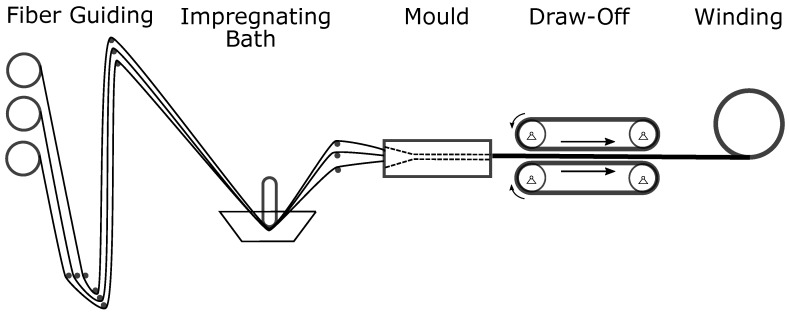
Schematic illustration of the pultrusion.

Pultrusion is a low-cost and good-quality manufacturing process frequently used for medium to high-volume commercial production. Parts with low geometrical complexity, especially prismatic parts with constant cross-section, can be manufactured in an endless manner with various reinforcing fibers and matrix systems. Figure 1 shows the steps of the pultrusion used in this study. The process starts with raw material in the form of endless fiber material on one end of the machinery. The fibers are then impregnated with the resin and guided into a closed mould. In the mould, the impregnated fibers are cured at elevated temperatures and receive their final shape. After the mould, grippers pull the material at relatively low speeds such as 1 m $\min^{-1}$. Lastly, the part is usually cut with a saw to the desired length. In this case, the finished material can simply be wound up into a roll because the part is very thin. Due to its focus on cost-sensitive applications, cure monitoring can attractively be used to increase the throughput of the machinery while simultaneously providing high quality parts with complete polymerization [8]. The relative movement of the fibers and the mould combined with high fiber volume ratios result in a highly abrasive environment. Mould design guidelines therefore generally recommend to use hardened steel or a chrome plating to provide a long working life [9]. Cure monitoring of the pultrusion therefore cannot rely on a direct contact between the sensor and the material since most sensors will be abraded very quickly. This work therefore analyzes ultrasonic cure monitoring techniques due to their inherent capability to allow for a measurement without direct contact of the sensor to the material.

Ultrasonic cure monitoring has been widely applied for different carbon fiber production technologies. Two parameters are generally observed and evaluated, namely the time-of-flight of a propagating wave and the amplitude attenuation characteristics [10,11,12,13,14]. Maffezzoli et al. [11] compared values obtained by ultrasonic techniques to those obtained from DSC. The authors used a 10 Mhz wavelet excitation for a pulse echo technique. When comparing acoustic and DSC results, the authors found that the point of gelation of the epoxy always occurs in between the maximum of signal attenuation and the onset of velocity increase. The authors concluded that, while good results were obtained, a statistical approach is needed to provide an exact correlation between the ultrasonic velocity and the degree of reaction. Mc Hugh [12] provided a good overview of the state of research in cure monitoring in his dissertation. He used commercial ultrasonic transducers and found a good match between the time-of-flight measurements and more common techniques such as rheometry. Lionetto et al. [13] analyzed a different experimental setup. The authors have been able to measure the changing sound velocity within epoxy by introducing air-coupled ultrasound, finding an acceptable agreement between their measurements and contact based approaches. Liebers et al. [14,15] analyzed a novel idea for ultrasonic transducers. The authors argued that the multiple reflective sites in commercial ultrasonic transducers limit the achievable signal strength. They therefore analyzed a setup, where the piezoelectric ceramics are directly glued onto the mould itself and found that this method provides easy integration, high signal strength and reliable measurements.

The available literature generally attempts to apply ultrasonic techniques to relatively thick specimens (2 mm [11], 2 mm [14], 18 mm [15], 2.3 mm [16]) and to plate-like objects with a large area. Very thin cross sections, such as 7 mm × 0.5 mm, have not yet been considered to be monitored with ultrasonic techniques. For these small cross-sections, the longitudinal wavelengths of the signal are approximately as large as or even larger than the geometrical dimensions of the mould. The potential disturbances have not yet been addressed in the literature of cure monitoring. On the one hand, thin cross sections result in comparably small time shifts of less than 100 ns that have to be considered when choosing the experimental hardware and could provide difficulties in signal analysis. On the other hand, the small width of the cross section could lead to interference patterns that could again hamper the signal analysis. All of these challenges are addressed in this article and some insight is presented on how to deal with these difficulties. This article furthermore proposes to apply pulse ultrasonic cure monitoring in pultrusion processes as a novel method to observe polymerization in this application. While multiple articles are available on pulse ultrasonic cure monitoring in prepreg and Resin Transfer Moulding (RTM) processes, the distinct challenges in the pultrusion process have not yet been discussed.

## 2. Materials and Methods

**Figure 2:**
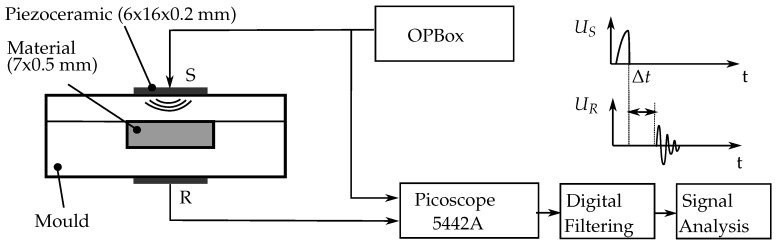
Measurement chain of the ultrasonic experiments applied in this paper.

Piezoelectric discs with a diameter of 16 mm and a thickness of 0.2 mm are used for both excitation and measurement in the experiments. A one-channel pulser and receiver box (OPBOX 2.1, Optel, Wrocław, Poland) is used as impulse generator for the actuation piezoceramic. The signal generated by the receiving piezoceramic is measured using a digital oscilloscope (Picoscope 5442A, Pico Technology, Cambridgeshire, UK). The maximum achievable time resolution of 4 ns is used for data acquisition. For the analysis of the received signal, a dedicated Python script automatically calculates the time-of-flight and amplitude attenuation. The exact methods for signal analysis are discussed in Section 3. The Measurement chain is summarized schematically in Figure 2.

**Figure 3:**
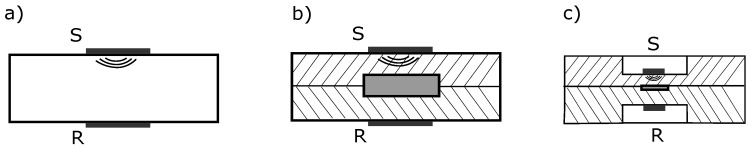
Mould geometries used in this paper. (**a**) A monolithic aluminum piece, (**b**) An aluminum tool with a large cross section of 15 mm × 2 mm, (**c**) A pultrusion tool with a small cross section of 7 mm × 0.5 mm.

Various types of adhesive material have been analyzed for their applicability for mounting the sensors on the mould, namely cyanoacrylate, silicone, and three different types of epoxy adhesive. When used in pultrusion, various boundary conditions must be taken into account for the adhesive. The temperature stability in particular is a critical factor. Liebers et al. [15] proposed in their work that RTM6 (Hexcel Corporation, Stamford, CT, USA) is a suitable adhesive for this. All measurements at elevated temperatures therefore use RTM6 as a mounting adhesive. Furthermore, the three different types of moulds displayed in Figure 3 are investigated, namely a monolithic aluminum piece, an aluminum mould with large cure cross section and a steel pultrusion tool with a small cure cross section. Two different epoxy based matrix systems are analyzed with the ultrasonic cure monitoring techniques. Initial experiments are carried out with the 5-min Epoxy from the company R&G (R&G Faserverbundwerkstoffe GmbH, Waldenbuch, Germany) due to its quick reaction time. Further experiments are conducted with the epoxy system RIMR426 (Lange+Ritter GmbH, Gerlingen, Germany) with the hardener RIMH435. This epoxy combination is referred to as RIM-epoxy in this article.

## 3. Results

### 3.1. Principle Analysis of the Experimental Setup

**Figure 4:**
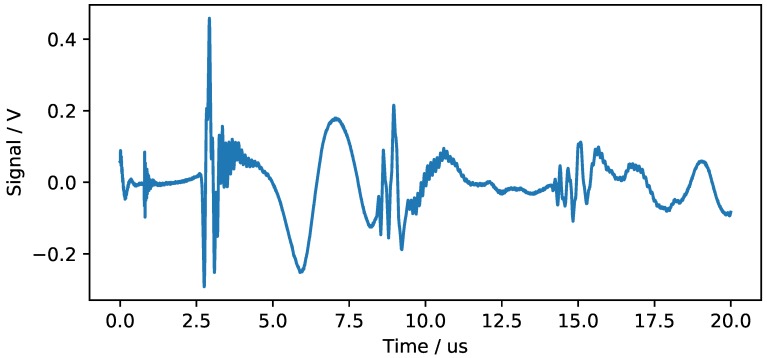
Typical signal received at the sensor for a pulse excitation at the opposite end.

**Figure 5:**
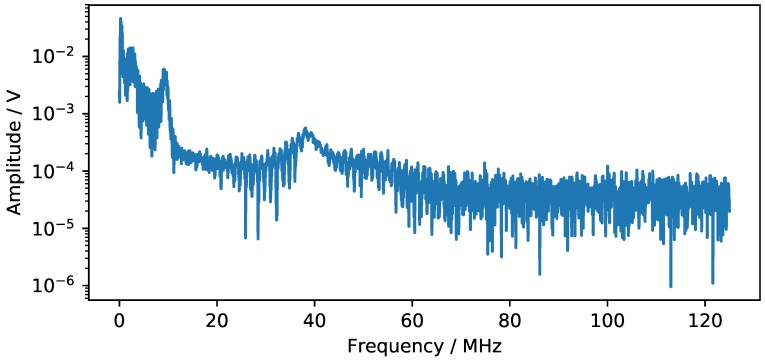
Frequency spectrum of the signal displayed in Figure 4.

In order to test and validate the described measurement chain, experiments were carried out on a monolithic piece of aluminum with a thickness of 18 mm as shown in Figure 3a. Two piezoceramics are mounted on opposite sides. Taking into account the fact that literature values for the longitudinal speed of sound in aluminum are generally around 6300 m $s^{-1}$, 18 mm should be passed in approximately 2.9 μs. Figure 4 and Figure 5 show typical results of the time data and the frequency spectrum acquired in this experiment. At t = 0, the transmitter pulse is triggered on its falling edge. A significant signal amplitude is observed at the beginning of the measurement. This cannot be attributed to a mechanical wave, as a signal delay of 2.9 μs would be expected. The phenomenon is due to the fact that both grounding planes of the piezoceramics are connected to the aluminum tool. Thus, electrical effects can interfere with the measurement immediately after a strong electrical impulse has been generated. Since this effect is not the aim of the study, such initial effects are neglected in the evaluations. After about 0.8 μs, another pattern is measured that resembles a typical exponential decay curve. The occurrence of this pattern is due to uninsulated electric cables and is also filtered out. After approximately 2.7 μs, a reproducible peak in the signal is measured. This value seems to be in reasonably good agreement to the literature value of 2.9 μs.

### 3.2. Principle Analysis of the Ultrasonic Cure Monitoring Technique

Subsequent experiments are conducted on an experimental tool, where pure epoxy with a cross section of 2 mm × 20 mm is cured between two 5 mm thick aluminum pieces. The speed of sound for longitudinal waves within a typical epoxy rises during polymerization from values in the range of 1500–2100 $m\;s^{-1}$, as reported in [17]. A simple calculation gives an expected time-of-flight between 2.6 μs and 3.2 μs. For the baseline experiments, a fast curing epoxy (5 min epoxy R&G) was mixed in a volume ratio of 1:1 and cured within the test mould at room temperature.

**Figure 6:**
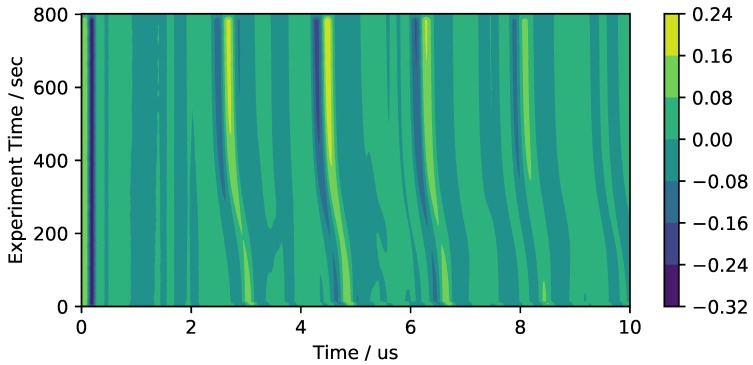
Colored contour diagram of the received signal during experiments on a large cross section.

The results of this experiment are visualized with the contour plot displayed in Figure 6. The *x*-axis shows the individual run time after the excitation pulse is triggered. The *y*-axis shows the experiment time after the mould is closed. The voltage generated by the piezoceramic is visualized by the different colors. Time shifts due to the curing epoxy can therefore be observed as an overall shift of the signal in the direction of the *y*-axis. This phenomenon can be easily observed in the acquired data. The first clearly shifting signal peak starts at approximately 3 μs at the beginning of the experiment and shifts to approximately 2.7 μs when the epoxy is fully cured. These values seem to be in reasonably good agreement with the expected values of the first longitudinal wave traveling through the mould. It is therefore concluded that the experimental setup can be used as a tool for ultrasonic cure monitoring of thermosetting matrix systems.

### 3.3. Experiments on a Steel Pultrusion Tool with Pure Resin

The challenge in this experimental setup lies within the small cross section of 7 mm × 0.5 mm analyzed in this study. As a first insight, it is found to be of importance to adjust the dimensions of the piezoelectric actuators to the width of the cross section. Initial experiments were conducted with the same piezoceramics as used in previous experiments. In these experiments, a mechanical wave simply bypassed the small mould and therefore did not change its time-of-flight during epoxy cure significantly. Only a rather small portion of the signal passes through the epoxy. Overall, it proved difficult to separate both of these signal parts within the measured signal. Therefore, it is important to cut the piezoceramic to be smaller than the width of cross section and then apply it centered above the mould.

Two different methods for determining the shift in time-of-flight are investigated in this article.

Firstly, a peak detection algorithm which specifically evaluates the exact position of a signal peak is applied. Over the years, a variety of peak detection algorithms have been proposed [18]. Users of available peak detection algorithms must specify a number of parameters to obtain an acceptable result. In the algorithm (python peakutils. indexes) used in this work, two parameters have to be specified, a threshold value and a minimum distance between the peaks. Only peaks larger than the threshold values are evaluated and the minimum distance constraint is always satisfied by eliminating smaller peaks. Based on these parameters, the algorithm will return the most significant peaks in the signal. Throughout the experiments, it was found that these values must be fine-tuned in order to gain acceptable results. Once a set of working parameters is found, the position of a peak is traced in time. The accuracy of each peak position is further increased by interpolation with a second order polynomial.

Secondly, the sweeping cross correlation method is used in an effort to find a more reliable method of determining the time shift in signals. Instead of trying to evaluate a specific point in each signal, this method compares each time signal $f_{signal}$ with the previous time step $f_{reference}$. This comparison is evaluated through the cross correlation of both signals:

(1) $$C_{f_{r},f_{s}}\left(n\right)=\sum_{m=-\infty }^{\infty }f_{reference}\left(m\right)f_{signal}\left(m+n\right).$$

The maximum value of the cross correlation $C_{f_{r},f_{s}}\left(n\right)$ represents the relative time shift at which both signals are most similar. The position of the maximum in the cross correlation can therefore be used to derive the difference in time-of-flight of both signals. The maximum time resolution of a cross correlation is equal to the time resolution of the provided signals. In order to increase this resolution, a cubic spline is fitted through the data points. An interpolated time resolution of 0.8 ns was found to be a good trade-off between resolution and computing time. After calculating the cross correlation shift of each time step with its preceding time step, the total time-shift throughout the experiment can be calculated by adding up all previous values. This method is therefore not based on the intricate fine-tuning of peak-finding parameters and is perceived as rather insensitive to differences in experimental conditions. In this work, the time window for the algorithm is chosen in a way that only the first echo is analyzed with this method. For the example displayed in Figure 6, the time window between 2 μs and 4 μs would be chosen.

The signal attenuation is described by the maximum voltage amplitude in the analyzed time window in this work. Two major factors influence the magnitude of the wave registered at the sensor piezoceramic. Firstly, the acoustic impedance of the epoxy polymer changes during cure. This further alters the ratio of transmission and reflexion on every interface from the tool material to the epoxy. Furthermore, the damping characteristic of the epoxy itself changes during cure. Both of these influencing factors are always measured simultaneously in this experimental setup.

**Figure 7:**
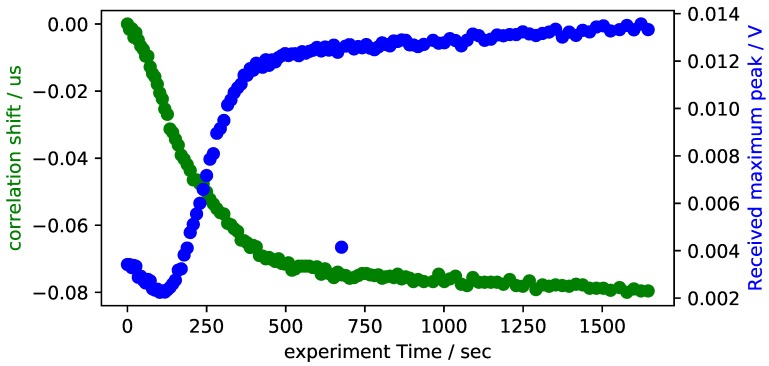
Signal attenuation and cross correlation shift of the experimental data with 5-min epoxy in a steel pultrusion tool.

In order to clear the signal from noise, a 2nd order digital bandpass is applied with a center frequency of 10 MHz and a bandwidth of 4 MHz. These values were chosen in a way that the resonance frequency of the piezoceramic is observed. Figure 7 shows the experimental results of the correlation analysis and attenuation. The general trend is very similar to the results obtained by other researchers [11,19]. Unlike other authors, however, the time shift falls rapidly from the beginning of the experiment. This is most likely due to the time it takes to assemble the mould, which can take up to 30 s after the epoxy is mixed. The epoxy is therefore already mixed for a significant fraction of the total time before the measurement started.

**Figure 8:**
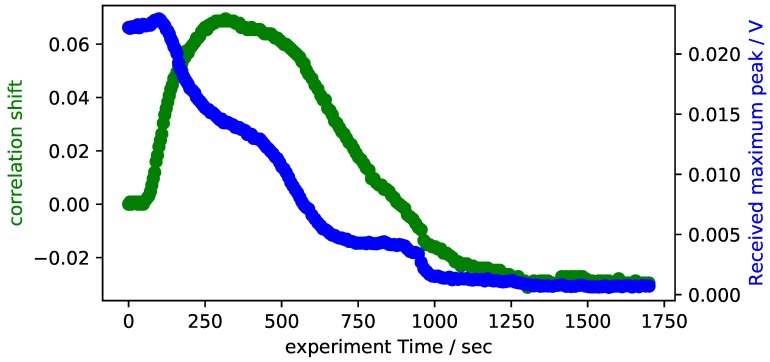
Signal attenuation and cross correlation shift of the experimental data with RIM-epoxy in a steel pultrusion tool.

Further experiments are conducted with the RIM-epoxy, which is better suited to the pultrusion process than the 5-min epoxy due to its smaller viscosity. Figure 8 shows the results of the experiments with RIM-epoxy. The epoxy is mixed at room temperature and then put into the bottom mould. The tool is assembled afterwards. After approximately 1 min, the tool is heated to 100 ${}^{\circ }C$ by means of integrated cartridge heaters. A number of differences can be observed when data is compared to the previously presented experiments with 5-min epoxy. First of all, the transmitted signal no longer increases after gelation occurs, but decreases monotonously throughout the experiment. Secondly, the correlation shift no longer falls monotonously, but now rises at first and only starts declining after approximately 250 s. The rise in correlation shift coincides with the heating of the tool. It is therefore attributed to most likely be due to a decrease of sound velocity in the epoxy due to heating. The subsequent decrease shows a total time shift of approximately 100 ns, which matches the expected time shift due to the polymerization of the epoxy.

### 3.4. Live Data Acquisition during the Pultrusion Process

**Figure 9:**
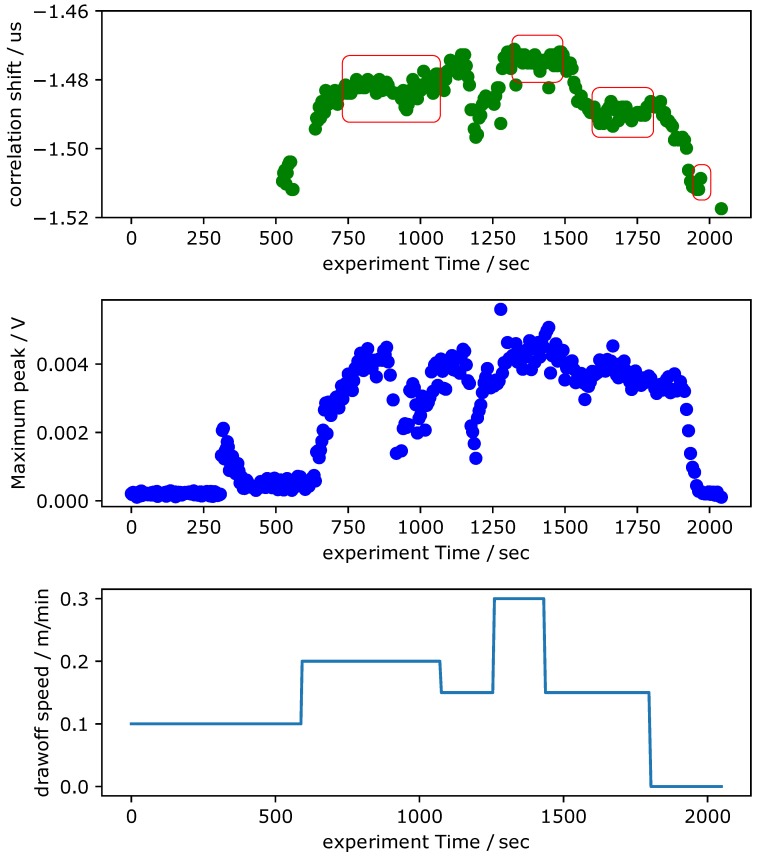
Correlation shift, received signal strength, and pull speed of the ultrasonic cure monitoring during the pultrusion process.

In this section, experiments are presented where the previously described ultrasonic cure monitoring techniques are used during the pultrusion. Experiments are carried out with the identical pultrusion tool used in the previous section. The tool is heated up homogeneously to 140 ${}^{\circ }C$. Five Toray HTS12 carbon fiber tows with 800 tex each are impregnated with the RIM-epoxy and then cured in a standard pultrusion process within the tool at variable pull speeds. The included tows and cross section lead to a fiber volume ratio of 64%. The raw data of the experiment is displayed in Figure 9. The top plots display the correlation shift and received signal strength measured 300 mm behind the entry of the tool. The bottom plot displays the current pull speed that was manually set for the pultrusion machine. The pull speed is varied between 0.1 m $\min^{-1}$ and 0.3 m $\min^{-1}$ during the experiment and stopped after approximately 1800 s. A number of points can be observed in these results. The first impregnated fibers reach the sensor after approximately 300 s. No coherent correlation shift can therefore be provided before this time. Once impregnated fibers reach the sensor, some reproducible results can be reported. This is regarded as proof that a signal transmission is achievable within the pultrusion process. The experimental data is comprehensively discussed in the following chapter.

## 4. Discussion

**Figure 10:**
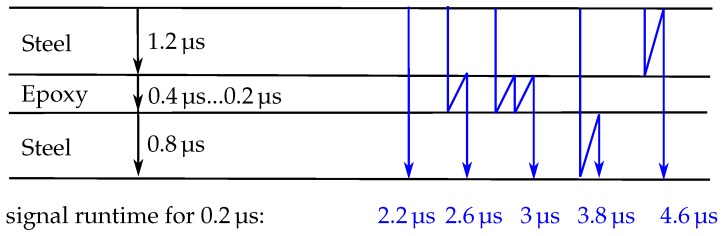
Reflective sites, signal paths and resulting signal time-of-flights for a mechanical wave in the closed mould process analyzed in this work.

The experimental results prove the basic working hypothesis that pulse ultrasonic monitoring techniques can be applied to a very thin cross section within a pultrusion tool. The sweeping cross correlation method generates very reliable signal time shifts even in measurements where the amplitude of the signal varies significantly over long time periods. The time shifts shown in this work are plausible and prove the working principle of the measurement chain. The evaluation of the signal attenuation shows mixed results. For the room temperature experiments with 5-min-epoxy, plausible results were obtained that match values reported in the literature. The RIM-epoxy system does not show similar attenuation mechanics. A closer analysis of the signal change over time shows that this could partly be due to interference patterns. We assume that the acoustic attenuation of the 5-min-epoxy is significantly larger than that of the RIM-epoxy based on its much larger viscosity. This simplifies the true mechanics of sound attenuation in liquids significantly [20], especially when the large frequencies analyzed in this work are considered. The assumption is nevertheless supported by the experimental data measured in these experiments. Please consider the potential signal runtimes displayed in Figure 10: If the signal attenuation of an epoxy is large, the secondary reflections within the epoxy have a rather small influence. A small signal attenuation on the other hand would result in significant interference on a small time scale of multiples of $2T_{Epoxy}$. In the 5-min-epoxy measurements, only the larger timescale interference due to reflections within the steel are observed. The RIM-epoxy measurements, however, do show significant interference changes in the signal on the smaller time scale. While these arguments do not completely explain the drop in received amplitude, they do show some major difficulties that arise when the amplitude of the received signal is analyzed in cure monitoring experiments with a very thin test specimen. As a first conclusion, the time-of-flight calculation through sweeping cross correlation is regarded to be the most promising analysis tool for cure monitoring of thin cross sections. It would, however, be interesting to see if the forming of these interference patterns could be exploited for a cure monitoring algorithm. This could potentially result in a much more sensitive analysis tool than the algorithms applied in this work.

When the tools are used in the pultrusion process, some process-specific factors have to be kept in mind. The epoxy is no longer curing at one specific place in the mould. Instead, the epoxy is now being pulled through the tool by the fibers and cures gradually from entry to exit. When the pull speed of the pultrusion is low, it is possible that the epoxy is fully cured after only a short distance inside of the tool—when the speed is high, it is possible that the dwell time at elevated temperatures of each epoxy volume is not sufficient to allow a complete cure at all.

Furthermore, the balance between thermal expansion and cure shrinkage results in a complex pressure profile in the pultrusion tool [8] (p. 263). It is well established that the pultruded part detaches from the surfaces of the tool due to epoxy shrinkage after vitrification occurs [21,22]. When this happens, it can be expected that no signal can traverse through the pultruded part itself. The large mismatch in acoustic impedance between the steel tool and air does not allow for a significant part of the wave to be transmitted. The experimental results show that the frequency spectrum of the received signal varies significantly over time. The characteristic peak at 10 MHz is only observed for comparably large pull speeds. This peak was previously attributed to be due to the resonance frequency of the sensor. It is possible that the existence of the 10 Mhz peak could be used as evidence for an acoustic coupling of the pultruded part to the mould. The wavelength λ of a 10 MHz wave in a steel tool is much smaller than the width of the actuator. It is well known that the directionality of acoustic emissions depends on the ratio of actuator dimensions *b* and wavelength λ [23]. Large ratios of $\frac{b}{\lambda }$ result in a highly directional acoustic emission. It is therefore recommended to strategically use piezoceramics with large fundamental Eigenfrequency and take advantage of the directionality of acoustic emissions at high frequencies for analyzing small cross sections.

**Figure 11:**
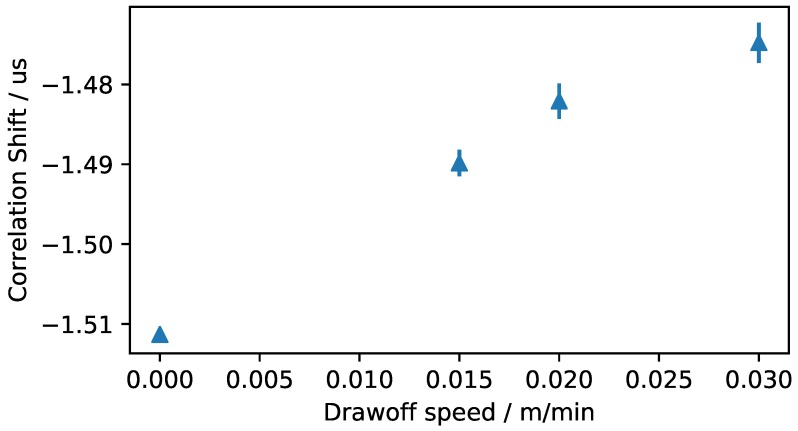
Connection of the Correlation Shift value in steady state condition and the pull speed during the pultrusion experiments.

The acquired data displayed in Figure 9 shows some general trends. First of all, no coherent correlation shift is presented before 500 s. The first coated fibers arrive at the sensor after approximately 300 s. Between 300 s and 500 s, only a small signal is transmitted. The received signal is generally higher when the pull speed is large. This is coherent with the previous experiments with the RIM-epoxy system, since the epoxy under the sensor is less polymerized and therefore more signal can be transferred. The fact that the time shift is also larger for higher speeds supports the interpretation of the given data. After approximately 850 s, the signal strength drops without a change in the pull speed. This could be due to insufficient impregnation of the fibers, but no definite interpretation can be presented at this point. It would be interesting, however, to further analyze this behavior and potentially exploit it to allow for a quality control. When the speed is dropped twice after approximately 1400 s, both correlation shift and signal strength show a clear drop indicating that the epoxy under the sensor is becoming more and more polymerized. The dependency of correlation shift signal and and pull speed is displayed in Figure 11. When the pull speed of the pultrusion changes, the signal propagation time does not change immediately because an epoxy volume that has not yet reached the sensor has cured under two different pull speeds. A steady state can only be achieved if an epoxy volume has been cured at a constant pull speed. Figure 11 therefore shows the mean value and standard deviation of the steady state correlation shifts for different pull speeds. The time segments interpreted as steady state are marked in Figure 9 in red boxes. Figure 11 clearly shows that the signal propagation time increases with increasing pull speed. This observation is regarded as evidence of the ability to monitor epoxy curing in the pultrusion process by analyzing the time of flight of a mechanical wave. The signal attenuation characteristics measured in this experiment show a much weaker correlation to the pull speed and are therefore not recommended to be used for cure monitoring in pultrusion at this point.

## 5. Conclusions

The ultrasonic cure monitoring technique developed in this research is by no means limited to the pultrusion and can be applied to many manufacturing processes where a polymer cures or solidifies in a mould. When applied to other composite manufacturing processes such as RTM, integrated ultrasonic sensors can offer online information about curing state as well as about the current resin propagation during injection. In this paper, the applicability of the approach was demonstrated for thin cross sections up to 0.5 mm. This value does not seem to be a lower boundary and even thinner cross sections could be analyzed in future investigations. For very thick cross sections, the signal attenuation within the polymer could at some point impede the signal transmission. However, this does not appear to be a problem in practice, especially with an infusion epoxy system such as the RIM-epoxy system analyzed in part of this work.

Overall, the results of the pultrusion experiments show some plausible results that prove the general applicability of the approach. However, it is also clearly observable that noise is present in the data. Significant improvements have to be achieved to allow for a full ultrasonic cure monitoring system with this setup. When compared to a static cure monitoring such as displayed in Figure 7, pultrusion monitoring shows an overall larger noise level and some stochastically distributed signal interruptions. This could be due to the relative movement of the carbon fibers and the tool. Another possible source of the larger noise level is an insufficient acoustic coupling that could occur in a dynamic process such as pultrusion in random intervals. At the current state of investigation, no clear answer can be given to this challenge. Further research will be conducted to more thoroughly understand the ultrasonic cure monitoring of the pultrusion process. Overall, a larger set of experimental data is required to further verify the monitoring results during pultrusion. Specifically, further research will focus on the influence of tool temperature and pull speed on the pultrusion cure monitoring. Additionally, comparative studies with a second ultrasonic cure monitoring system that is being investigated within the authors’ research group will be performed. Pommer et al. [24] analyze an alternative cure monitoring technique that uses a resonant based technique instead of a pulse ultrasonic measurement. A comparative study is under development that will give further insights into the ultrasonic monitoring of the pultrusion process.

The pulse ultrasonic techniques could be significantly improved when the acoustic properties of the mould are taken into consideration. Reflection and absorption of the mechanical wave occurs due to the change in acoustic impedance at the boundaries within the pultrusion tool. Interference patterns are clearly visible in the observed data; however, they are not yet analyzed for cure monitoring. By modeling these properties for a specific tool, a much more precise algorithm for cure monitoring could be devised. Furthermore, the wavelet excitation, which could not be explored in this study since no amplifier was available, could be tried as an excitation instead of the pulse signal applied here. Due to the narrow frequency spectrum of a wavelet, it should be more simple and robust to measure the curing process within the noisy pultrusion environment.

## Abbreviations

The following abbreviations are used in this manuscript:

CFRP	Carbon Fiber Reinforced Plastics
DSC	Differential Scanning Calorimetry
FTIR	Fourier Transform Infrared Spectrometry
RTM	Resin Transfer Moulding
